# Defense Molecules of the Invasive Plant Species *Ageratum conyzoides*

**DOI:** 10.3390/molecules29194673

**Published:** 2024-10-01

**Authors:** Hisashi Kato-Noguchi, Midori Kato

**Affiliations:** Department of Applied Biological Science, Faculty of Agriculture, Kagawa University, Miki 761-0795, Kagawa, Japan

**Keywords:** allelochemical, herbivore, invasive species, natural enemy, nematode, pathogen, precocene, pyrrolizidine alkaloid

## Abstract

*Ageratum conyzoides* L. is native to Tropical America, and it has naturalized in many other tropical, subtropical, and temperate countries in South America, Central and Southern Africa, South and East Asia, Eastern Austria, and Europe. The population of the species has increased dramatically as an invasive alien species, and it causes significant problems in agriculture and natural ecosystems. The life history traits of *Ageratum conyzoides*, such as its short life cycle, early reproductive maturity, prolific seed production, and high adaptive ability to various environmental conditions, may contribute to its naturalization and increasing population. Possible evidence of the molecules involved in the defense of *Ageratum conyzoides* against its natural enemies, such as herbivore insects and fungal pathogens, and the allelochemicals involved in its competitive ability against neighboring plant species has been accumulated in the literature. The volatiles, essential oils, extracts, residues, and/or rhizosphere soil of *Ageratum conyzoides* show insecticidal, fungicidal, nematocidal, and allelopathic activity. The pyrrolizidine alkaloids lycopsamine and echinatine, found in the species, are highly toxic and show insecticidal activity. Benzopyran derivatives precocenes I and II show inhibitory activity against insect juvenile hormone biosynthesis and trichothecene mycotoxin biosynthesis. A mixture of volatiles emitted from *Ageratum conyzoides*, such as β-caryophyllene, β-bisabolene, and β-farnesene, may work as herbivore-induced plant volatiles, which are involved in the indirect defense function against herbivore insects. Flavonoids, such as nobiletin, eupalestin, 5′-methoxynobiletin, 5,6,7,3′,4′,5′-hexamethoxyflavone, and 5,6,8,3,4′,5′-hexamethoxyflavone, show inhibitory activity against the spore germination of pathogenic fungi. The benzoic acid and cinnamic acid derivatives found in the species, such as protocatechuic acid, gallic acid, *p*-coumaric acid, *p*-hydroxybenzoic acid, and ferulic acid, may act as allelopathic agents, causing the germination and growth inhibition of competitive plant species. These molecules produced by *Ageratum conyzoides* may act as defense molecules against its natural enemies and as allelochemicals against neighboring plant species, and they may contribute to the naturalization of the increasing population of *Ageratum conyzoides* in new habitats as an invasive plant species. This article presents the first review focusing on the defense function and allelopathy of *Ageratum conyzoides*.

## 1. Introduction

**Figure 1:**
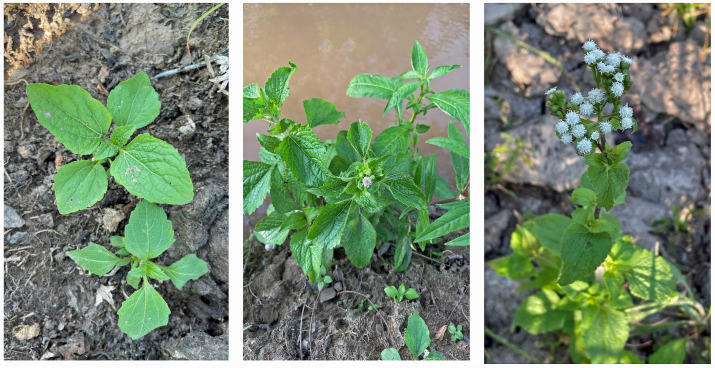
*Ageratum conyzoides*. Photos were kindly provided by Dr. Poonpaiboonpipat, T.

*Ageratum conyzoides* L., belonging to the family Asteraceae, is an annual or subshrub and grows to 20–150 cm in height. The stems are erect and round, covered with villi, and they branch well. The opposite leaves are simple, ovate, serrate, pubescent, 2–8 cm long, and 1–5 cm wide, with long petioles. It has a fibrous root system. The capitula are 4–6 cm in diameter, generated in panicles at the ends of the twigs, and a single capitulum contains 30–50 tubular florets. The corollas of the florets are white to mauve. The fruits are black and liner achenes, having aristate pappi [1,2,3,4] (Figure 1).

The native range of *Ageratum conyzoides* consists of Tropical America. The species is thought to have been introduced into different countries as an ornamental plant, but it has naturalized and spread in many tropical, subtropical, and temperate countries in South America, Central and Southern Africa, South and East Asia, Eastern Austria, and Europe [1,2,3,4,5]. Primary infestation may occur along road margins because the density of the species population is correlated with the distance from roads [2]. It was estimated that 40% of the geographical areas in the Eastern Ghats of India would be covered by *Ageratum conyzoides* by the end of 2100 [6].

The population of *Ageratum conyzoides* has been reported to have increased dramatically and it causes significant problems in agriculture in the introduced ranges. The infestation of *Ageratum conyzoides* has suppressed the production of more than 30 crops over 40 countries [6,7,8,9]. For example, the species reduced the production of direct-seed rice by 15–65%, soybean by 50–75%, maize by 15–65%, and groundnut by 45–70% [10]. *Ageratum conyzoides* also acts as a host for many crop diseases, such as okra enation leaf curl virus, capsicum chlorosis virus, cotton leaf curl virus, and tomato yellow leaf curl virus [11,12,13], and as a host of aphids that carry papaya ringspot virus [14]. The infestation of *Ageratum conyzoides* in grasslands reduced the production of grass fodder, causing a shortage in the fodder supply for livestock [15]. The infestation of *Ageratum conyzoides* has also been reported to significantly affect natural ecosystems. The species formed dense monocultural stands on forest floors and grasslands, reducing the species diversity by 32%, fresh biomass by 40%, and dry biomass by 49% in the introduced ranges [16]. Its infestation has been reported to threaten the survival of protective indigenous plant species on the Hawaiian islands, including *Isodendrion longifolium* and *Brighamia insignis* [4,17].

Its life history traits, such as its high growth rate, high reproduction rate, and high adaptivity, including phenotypic plasticity, contribute to the naturalization of this invasive plant species and to increasing its population in the introduced ranges [18,19,20,21,22]. *Ageratum conyzoides* has a short life cycle and early reproductive maturity. The species can complete its life cycle in less than 2 months, and it bears flowers when two leaves expand [23,24]. *Ageratum conyzoides* produces two generations a year under favorable growth conditions [24]. The species produces 40,000–95,000 seeds per plant [4,15,24]. The seeds are small and lightweight, and dispersed through water and wind, the attachment of the aristate pappus to stick to animals and human clothes, and the contaminant in crops and soil [3,4,5]. The average dispersal distance was recorded to be 2.4 km per year [25]. The seeds did not show any marked dormancy, and half of the seeds germinated [15,23,24,26,27,28,29].

*Ageratum conyzoides* thrives in open areas with high humidity and high soil fertility and at temperatures ranging between 20 °C and 25 °C [4,5]. Its chromosome number was reported to be 2n = 20 or 40 [1,4,30]. The species has great morphological variety and is highly adaptive to different moisture and temperature conditions and shade conditions [31]. The species has survived at temperatures between 15 °C and 30 °C [5]. The species was found in mountain areas at up to 1800 m elevation [28,32]. *Ageratum conyzoides* also maintains its dense population under dry and shaded conditions [33,34,35]. The species has infested protective forests, in which the forest floor was relatively dark, and destroyed the community of the native undergrowth species [34,35]. These observations suggest that the life history traits of *Ageratum conyzoides*, such as its short life cycle and early reproductive maturity, prolific seed production, and high adaptivity to various environmental conditions, may contribute to the invasiveness of the species.

Many of the invasive plant species are also reported to possess defense molecules, which are involved in defense functions against natural enemies, such as herbivores and pathogens, as well as allelochemicals involved in allelopathy against competitive plant species [19,20,36,37,38,39]. These compounds may also contribute to the invasiveness of *Ageratum conyzoides*. However, there has been no review article focusing on the defense molecules, including allelochemicals, of *Ageratum conyzoides* involved in such functions. This work provides an overview of the defense responses and allelopathy of the species, and the compounds involved in its defense functions. The action mechanisms of the molecules involved in the defense functions are also discussed. The literature has been searched using a combination of the predominant online search engines, i.e., Scopus, ScienceDirect, and Google Scholar, and all possible combinations of *Ageratum conyzoides* with the following terms: botany, biology, habitat, reproduction, adaptively, plasticity, invasiveness, impact, natural enemy, insecticidal activity, fungicidal activity, nematode, symbiosis, rhizobium, allelopathy, allelochemical, pharmacology, and second metabolite.

## 2. Defense Molecules against Herbivore Insects

One of the essential factors for plant species to survive invasion is their defense ability against herbivore insects as natural enemies. Herbivore insects sometimes cause significant damage to plant growth, development, and regeneration [40,41,42]. Therefore, some plant species have developed a chemical defense strategy against their natural enemies [19,20,43,44].

Aqueous extracts of *Ageratum conyzoides* stems and leaves increased the mortality of an adult polyphagous grasshopper (*Zonocerus variegatus*) [45]. Hexane extracts of *Ageratum conyzoides* leaves also increased the mortality of the adult insects of *Diaphania hyalinata*, *Musca domestica*, *Periplaneta americana*, and *Rhyzopertha dominica* [46]. The whole plant extracts of *Ageratum conyzoides*, using aqueous solutions, methanol, and other organic solvents, showed insecticidal activity against several crop pest insects, such as a stalk borer (*Chilo partelus*) [47], a rice weevil (*Sitophilus oryza*), a rice bug (*Leptocorisa chinensis*) [48], and a mosquito (*Anopheles gambiae*), which is the most important vector of malaria [49]. The essential oil of *Ageratum conyzoides* also showed insecticidal activity against a crop grain insect (*Tribolium castaneum*) [50] and inhibitory activity regarding the metamorphosis of a cowpea weevil (*Callosobruche naculatus*) [51]. The essential oil showed ovicidal activity and reduced the fertility of a cotton strainer (*Dysdercus angulatus*) [48].

**Figure 2:**
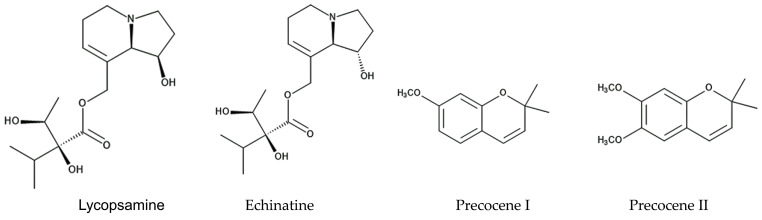
The compounds involved in the insecticidal activity of *Ageratum conyzoides.*

Two isomeric pyrrolizine alkaloids, lycopsamine and echinatine, were found in extracts of *Ageratum conyzoides* [52]. Pyrrolizidine alkaloids consist of a necine base and a double five-membered ring with a nitrogen atom in the middle, esterified with mono- or dicarboxylic acids, called a necic acid [53]. Pyrrolizidine alkaloids have been found in more than 300 different compounds in the plant families of Asteraceae, Boraginaceae, Fabaceae, and Orchidaceae [54]. These compounds are synthesized from L-arginine, and the specific intermediate is a homospermidine (polyamine). Pyrrolizidine alkaloid *N*-oxides are some of the primary products of pyrrolizidine alkaloid biosynthesis [55]. These plant species may produce these pyrrolizidine alkaloids as chemical defense agents against herbivores, such as insects and mammals [56,57,58,59]. The compounds are highly toxic, showing highly hepatotoxic, genotoxic, cytotoxic, tumorigenic, and neurotoxic activity. After absorption by insects and mammals, the first step in the activation of pyrrolizidine alkaloids is dehydrogenation catalyzed by cytochrome P450 monooxygenases [60,61], and the activated compounds interrupt several types of metabolism in the cell functions of these insects and mammals [62,63]. Therefore, the pyrrolizidine alkaloids in *Ageratum conyzoides* may be involved in the insecticidal activity caused by the extracts and essential oil of the species, as described above, and contribute to the protection of the species from herbivore attacks (Figure 2).

However, certain specialist herbivores have evolved tolerance to pyrrolizidine alkaloids. These specialists accumulate and store pyrrolizidine alkaloids in certain organs. The accumulated pyrrolizidine alkaloids are used for protection from their predators as poison and as precursors to synthesize mating pheromones. Some of these insects also transfer the pyrrolizidine alkaloids to their eggs for the protection of their offspring [53,64,65]. However, *Ageratum conyzoides* may seldom meet these specialist insects in its introduced ranges, because there may be no such coevolutionary history between these insects and *Ageratum conyzoides* in the introduced ranges.

The extracts and essential oil of *Ageratum conyzoides* were reported to contain two benzopyran derivatives, precocene I and precocene II (formerly named ageratochromene) [48,66,67]. These compounds are toxic and have shown anti-juvenile hormone activity, such as the inhibition of the reproduction of a bean beetle (*Epilachna varivestis*), the induction of diapause in a potato beetle (*Leptinotarsa decenlineata*) [66], and the inhibition of the metamorphosis of a moth (*Spodoptera manuritta*) [68]. The juvenile hormone is known to control several aspects of insect development, such as reproduction, diapause, and metamorphosis [69]. Precocene II was reported to inhibit the biosynthesis of the juvenile hormone [70]. In addition, precocene II was reported to cause morphological abnormalities in the pupae development of a crop pest beetle (*Epilachna vigintioctopunctata)* [71] and to interrupt mitochondrial function in rat cells [72]. These observations suggest that precocene I and precocene II may suppress insect growth and development due to the interruption of juvenile hormone biosynthesis and contribute to protection from herbivore insect attacks as defense molecules (Figure 2).

**Figure 3:**
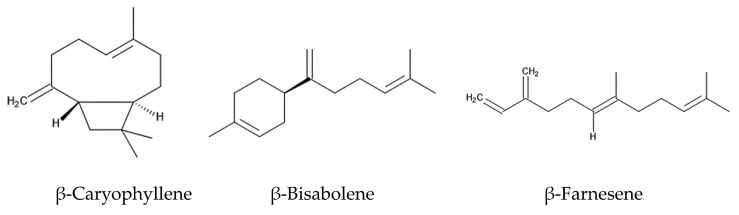
The compounds that act as HIPVs involved in indirect defense function.

The intercropping of *Ageratum conyzoides* in citrus orchards increased the population of a predator mite, *Amblyseius newsami*, which hunts for a herbivore mite, *Panonychus citri*. *Panonychus citri* is the natural enemy of citrus and reduces citrus production significantly [68,73]. *Ageratum conyzoides* emits a mixture of volatiles, such as precocenes I and II, and three sesquiterpenes: β-caryophyllene, β-bisabolene, and β-farnesene (Figure 3). The concentrations of these volatiles in the air of *Ageratum conyzoides*-intercropping citrus orchards were greater than those in non-intercropping citrus orchards [67].

When herbivore insects attack, certain plants emit a mixture of volatiles consisting of different chemical classes, called herbivore-induced plant volatiles (HIPVs) [70]. HIPVs stimulate predators to hunt herbivores as their prey. The predator insects sense HIPVs via the olfactory sensilla located on their antennae [74]. The responses of predator insects to HIPVs vary among predator species, and only a particular mixture of HIPVs (chemical competition and concentration) serve as signals for specific insects [75]. Then, the sensorial functions trigger the hunting behavior of these insects against the herbivores. HIPVs are considered to be involved in the indirect defense function of plants against herbivores [74,75]. The essential oil of *Ageratum conyzoides* and a volatile mixture of precocenes I and II, β-caryophyllene, β-bisabolene, and β-farnesene attracted *Amblyseius newsami* [67]. Therefore, the volatile mixture emitted from *Ageratum conyzoides* may serve as HIPVs involved in indirect defense function. β-Farnesene is known to act as a HIPV in several other plant species [75].

## 3. Defense Molecules against Nematodes

Plant-parasitic nematodes, such as root-knot nematodes *Meloidogyne* spp., are some of the major plant pathogens [76,77]. The host range of *Meloidogyne* spp. is wide, and their parasitism causes significant growth retardation in the host plant species. The nematodes creates galls in the plant roots and reduce the photosynthates and nutrients available to their host plants, leading to the loss of plant vigor and defense capabilities against other pathogen attacks [78,79,80]. Aqueous extracts of *Ageratum conyzoides* leaves increased the mortality of *Meloidogyne incognita* [81] and *Meloidogyne javanica* [82]. Its aqueous leaf extracts also suppressed the parasitic gall formation of *Meloidogyne incognita* [83]. Although the active compounds in the extracts have not yet been determined, these observations suggest that *Ageratum conyzoides* may possess certain compounds that have nematicidal activity. As described in Section 2, *Ageratum conyzoides* contains pyrrolizidine alkaloids, which are highly toxic to insects and mammals [60,61]. Therefore, these pyrrolizidine alkaloids may be involved in the nematicidal activity of the species.

## 4. Defense Molecules against Fungal Pathogens

**Figure 4:**
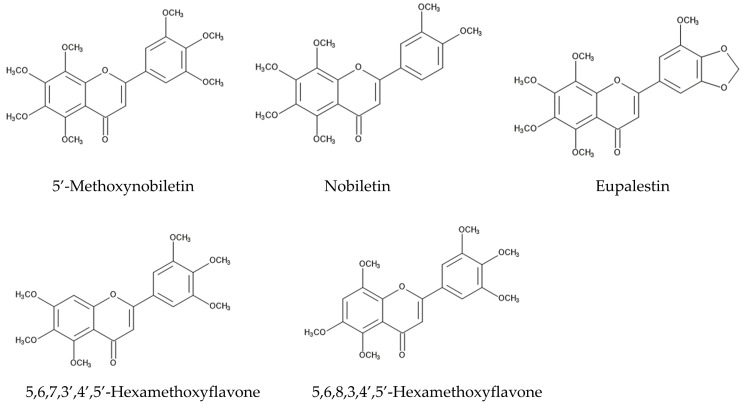
The compounds involved in the fungicidal activity of *Ageratum conyzoides.*

The defense ability against fungal pathogens is one of the essential factors for plants to survive an invasion. Some *Fusarium* spp. are fungal plant pathogens, causing diseases such as rot, blights, cankers, and wilts in the host plant tissue [84,85,86]. *Fusarium* also produces a number of mycotoxins, such as trichothecenes and fumonisins [87,88]. Aqueous *n*-hexane and methanol extracts of whole plants of *Ageratum conyzoides* suppressed the growth of *Fusarium solani*, which causes rot and wilt diseases [89]. Methanol extracts of the aboveground parts of *Ageratum conyzoides* suppressed the growth of *Fusarium oxysporum*, which causes blight and wilt diseases [90]. In addition, extracts of the aerial parts of *Ageratum conyzoides* inhibited the growth of a rice blast fungus, *Pyricularia oryzae*, and a sugar beet root rot fungus, *Rhizoctonia solani*. Precocene II and four flavonoids, nobiletin, 5′-methoxynobiletin, eupalestin, and 5,6,7,3′,4′,5′-hexamethoxyflavone, were identified in the extracts as the active compounds, and the inhibitory activity of precocene II was the highest among them [91] (Figure 4).

The intercropping of *Ageratum conyzoides* in citrus orchards decreased the populations of the soil-pathogenic fungi *Phytophthora citrophthora*, *Pythium aphanidermatum*, and *Fusarium solani*. Precocenes I and II and three flavonoids, 5′-methoxynobiletin (5,6,7,8,3,4′,5′-heptamethoxyflavone), 5,6,7,3′,4′,5′-hexamethoxyflavone, and 5,6,8,3,4′,5′-hexamethoxyflavone, were found in the soil where *Ageratum conyzoides* was intercropped. These compounds inhibited the spore germination of these pathogenic fungi [92]. These observations suggest that *Ageratum conyzoides* possesses antifungal activity and precocenes I and II, as well as the mentioned flavonoids, may be involved in this activity.

Precocenes I and II were reported to inhibit the production of trichothecene mycotoxin in a pathogenic fungus, *Fusarium graminearum*. The inhibitory activity of precocene II was much greater than that of precocene I [93,94]. Trichothecene is synthesized from farnesyl pyrophosphate, which is produced through the mevalonate pathway, and its synthesis is regulated by the TRI6 (trichothecene biosynthesis positive transcription factor) protein encoded by *Tri6* genes [95,96]. Precocenes II binds to a mitochondrial outer membrane protein and elevates the mitochondrial superoxide levels. The high levels of superoxide in mitochondria decrease the *Tri6* gene levels and TRI6 protein, resulting in the suppression of trichothecene production [95,96,97]. In addition, the insect juvenile hormone is also synthesized from farnesyl pyrophosphate in the corpus allatum cells of insects [98]. As described in Section 2, precocene II was reported to interrupt mitochondrial function [72] and to inhibit the biosynthesis of the juvenile hormone [70]. Therefore, precocene II may bind to the mitochondrial membrane proteins of the corpus allatum cells and interrupt juvenile hormone biosynthesis.

These observations suggest that precocenes I and II and these flavonoids may work as defense molecules against fungal pathogen attacks and help the invasion of *Ageratum conyzoides* into the introduced ranges.

## 5. Inhibitors for Symbiosis

When the whole plant residues of *Ageratum conyzoides* were mixed with soil, the soil suppressed the growth and nodulation of a leguminous plant chickpea (*Cicer arietinum*) [99]. Leguminous plants generally coexist with symbiotic rhizobia [100,101,102]. Rhizobium nodulation enhances the host plant’s performance through the supply of nitrogen and ammonium to the host plant [103,104]. *Ageratum conyzoides* may possess certain compounds that degrade the nodulation of nearby legume plants. A reduction in rhizobium nodulation weakens the ability of these legumes to perform nitrogen and ammonium acquisition, which may cause the growth suppression of these plant species. Some other invasive plant species were also reported to suppress the colonization of the rhizobia and arbuscular mycorrhiza of native plant species [105,106]. Certain flavonoids released from leguminous plant species are known to act as signals for the induction of the nodulation genes in rhizobia and the initiation of symbiosis [103,104]. The compounds in *Ageratum conyzoides* may reduce the rhizobium population and interfere with the flavonoid signals and/or nodulation, resulting in the interruption of the symbiosis between the legumes and rhizobia. However, there is no information available on the compounds involved in the interruption of this symbiosis. The identification of these compounds is necessary.

## 6. Defense Molecules against Neighboring Plants

Allelopathy is the plant-to-plant interaction in the local plant community, occurring through certain secondary metabolites defied as allelochemicals. The donor plant species produce and release allelochemicals into their neighboring environments, and these released allelochemicals suppress the germination, growth, development, and/or regeneration process of the receiver plant species. Subsequently, the donor plants gain a relatively large quantity of resources, such as light, water, and nutrients, in the local plant community [107,108,109,110]. The competitive ability of invasive plant species against indigenous plant species for resource acquisition is one of the most important factors for their success in the introduced ranges [19,20,111,112]. The allelopathic potential of invasive plant species against indigenous plant species is often reported to be high [113,114,115].

The inhibitory effects of certain allelochemicals in invasive plant species against competitive plant species are considered to be greater in the introduced ranges than in the native ranges of the invasive plant species. In their native ranges, the competitive plant species may have developed tolerance to these allelochemicals because of their coevolutionary history. However, in their introduced ranges, the competitive plant species may not have had an opportunity to acquire tolerance to these allelochemicals because they had not existed together before. Therefore, according to the novel weapons hypothesis, the allelochemicals released from invasive plant species are more effective on indigenous plant species in the introduced ranges and contribute to their invasiveness [36,111,112].

Allelochemicals are synthesized, stored in certain plant organs, and released into the neighboring environment through volatilization, root exudation, and the decomposition of plant residues in the rhizosphere soil [107,108,109,110]. Therefore, allelochemicals have been identified in the extracts of plant organs (leaves, stems, and roots), essential oils, volatiles, root exudates, and rhizosphere soil [116,117,118].

Aqueous extracts of *Ageratum conyzoides* leaves inhibited the germination and growth of *Parthenium hysterophorus* [119]. Acetone extracts of *Ageratum conyzoides* leaves and roots inhibited the germination and growth of *Oryza sativa* [120]. Acetone extracts of *Ageratum conyzoides* shoots (leaves and stems) inhibited the germination and growth of *Amaranthus caudatus*, *Digitaria sanguinalis*, and *Lactuca sativa* in an extract concentration-dependent manner [121]. Meanwhile, *n*-hexane and ethyl acetate extracts of *Ageratum conyzoides* leaves inhibited the growth of *Amaranthus spinosus*, and a major constituent in both extracts was precocene II [122,123]. These observations suggest that *Ageratum conyzoides* contains certain extractable allelochemicals, including precocene II.

The whole plant powder of *Ageratum conyzoides* incorporated into the soil inhibited the germination and growth of *Echinochloa crus-galli*, *Monochoria vaginalis*, and *Aeschynomene indica.* Coumalic acid, gallic acid, and benzoic acid were major constituents in the aqueous methanol extracts of *Ageratum conyzoides* whole plants [124]. When the root residues of *Ageratum conyzoides* were incorporated into soil, the soil suppressed the growth of *Oryza sativa* [125], and protocatechuic acid, *p*-coumaric acid, gallic acid, ferulic acid, and *p*-hydroxybenzoic acid were identified in the aqueous extracts of the soil as allelopathic agents [8]. The aqueous extracts of soil previously infested by *Ageratum conyzoides* inhibited the growth of *Triticum aestivum* [126]. The root exudates of *Ageratum conyzoides* suppressed the germination and growth of *Abelmoschus esculentus*, *Solanum lycopersicum*, *Phaseolus vulgaris*, *Zea mays*, *Cicer arietinum*, and *Cucumis sativus* [127]. These observations suggest that *Ageratum conyzoides* may contain certain allelochemicals, which are released into the rhizosphere soil through the root exudation and decomposition processes of plant residues. Protocatechuic acid, *p*-coumaric acid, gallic acid, ferulic acid, and *p*-hydroxybenzoic acid may be some of these allelochemicals.

The intact fresh leaves of *Ageratum conyzoides* and its essential oil inhibited the growth of *Cucumis sativus*, *Lolium ultiforum*, *Raphanus sativus*, *Phaseolus aureus*, *Triticum aestivum*, and *Lycapesicon* spp. in sealed bottles. Precocenes I and II and β-caryophyllene were found as active compounds [128]. This observation suggests that certain allelochemicals, including precocenes I and II and β-caryophyllene, may be released into the air through volatilization from *Ageratum conyzoides*.

Precocenes I and II elevate the mitochondrial superoxide levels in the cells of insects and fungi, leading to insecticidal activity and fungicidal activity, as described in Section 4. High levels of superoxide were also reported to cause allelopathic activity, such as germination and growth inhibition against several plant and alga species [129,130,131]. Therefore, the allelopathic activity of precocenes I and II may also be caused by the elevation of the superoxide levels in plant cells. Three sesquiterpenes, β-caryophyllene, β-bisabolene, and β-farnesene, emitted from *Ageratum conyzoides* showed allelopathic activity [132]. In addition, β-caryophyllene was identified in another invasive plant species, *Mikania micrantha* [133], and was considered to act as a allelochemical due to the elevation of the superoxide levels in the cells of the receiver plant species [134,135,136]. Therefore, these sesquiterpenes emitted from *Ageratum conyzoides* may induce allelopathic activity due to the elevation of the superoxide levels in the cells of the receiver plant species.

Soil samples obtained from *Ageratum conyzoides*-intercropped citrus orchards suppressed the growth of *Bidens pilosa*, *Digitaria sanguinalis*, and *Cyperus difformis*. Precocene II and three flavonoids, 5′-methozynobiletin, 5,6,7,3′,4′,5′-heptamethoxyflavone, and 5,6,8,3,4′,5-hexamethoxyflavone, were identified in citrus orchard soil as allelopathic agents [92] (Figure 4). Flavonoids are polyphenolic secondary metabolites synthesized from phenylalanine through chalcone. Many flavonoids are reported to have anti-fungal, anti-herbivore, anti-bacterial, and allelopathic activity [137,138,139,140]. *Ageratum conyzoides* is rich in polyoxygenated flavonoids [141]. Therefore, some of these identified flavonoids and precocene II in *Ageratum conyzoides* may be released into the rhizosphere soil through the decomposition of plant residues and act as allelopathic agents. However, the molecular targets of the flavonoids in plant cells are unknown.

**Figure 5:**
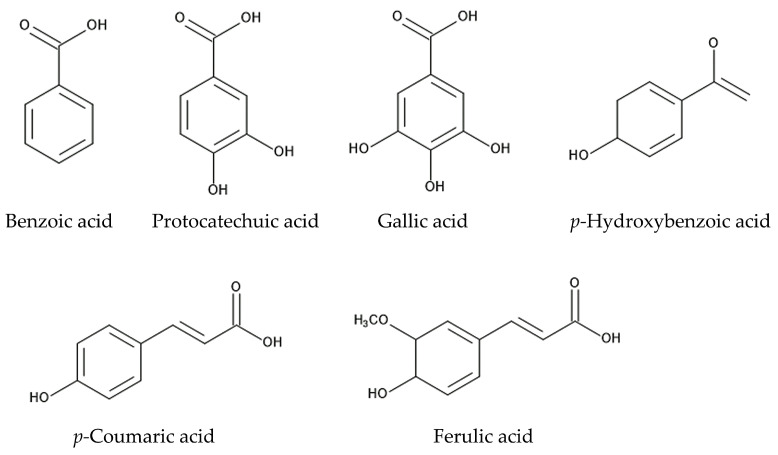
The compounds involved in the allelopathy of *Ageratum conyzoides.*

Benzoic acid and cinnamic acid derivatives, such as protocatechuic acid, gallic acid, *p*-coumaric acid, *p*-hydroxybenzoic acid, and ferulic acid, were identified in extracts of the roots, leaves, and stems of *Ageratum conyzoides* and/or in soil mixed with its root residues [8,124,125]. Cinnamic acid and its derivatives are synthesized via the shikimic acid pathway from phenylalanine [142,143]. These compounds have been identified in a wide range of plant extracts, plant residues, and plant rhizosphere soil. The involvement of benzoic acid and cinnamic acid derivatives in allelopathy and the mechanisms of their allelopathic action have been investigated in other plant species [144,145,146]. These compounds cause structural alterations in the plasma membrane lipids and proteins of plant cells and reduce the transmembrane electrochemical potential, which causes the depolarization of the membranes. The depolarization of the membranes induces the nonspecific efflux of both cations and anions, including phosphate, potassium, magnesium, and nitrate ions, and affects the water balance in the cells. These compounds also interrupt the activity of various enzymes involved in several types of metabolism, such as photosynthesis, respiration, phytohormone synthesis, protein synthesis, and the synthesis of other secondary metabolites, and they affect plant cell division, growth, and development [144,145,146,147]. Therefore, benzoic acid, protocatechuic acid, gallic acid, *p*-coumaric acid, *p*-hydroxybenzoic acid, and ferulic acid may affect the plasma membrane structure and certain enzymes’ activity and act as allelopathic agents (Figure 5).

These allelochemicals identified in *Ageratum conyzoides* may contribute to the suppression of the germination and growth of indigenous plant species and increase the competitive ability of *Ageratum conyzoides* for the acquisition of nutrients, water, and light in the introduced ranges.

## 7. Contributions of Defense Molecules to Invasive Traits of *Ageratum conyzoides*

**Figure 6:**
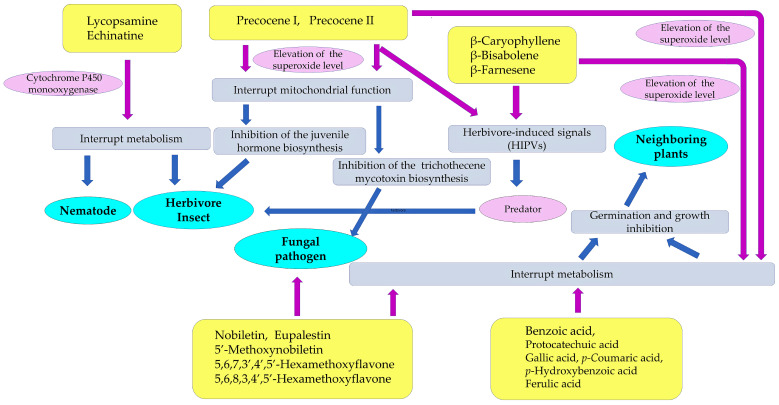
Defense molecules involved in the invasive abilities of *Ageratum conyzoides*. These compounds act as nematicidal, insecticidal, fungicidal, and allelopathic agents of *Ageratum conyzoides*. Purple arrow: direct action; blue arrow: secondary and tertiary action.

*Ageratum conyzoides* produces several defense molecules against insects, nematodes, fungal pathogens, and competitive neighboring plant species. Among them, precocenes I and II showed insecticidal activity through the inhibition of insect juvenile hormone biosynthesis [70]. Precocenes I and II also have fungicidal activity [92], and they exhibit inhibitory activity regarding trichothecene mycotoxin biosynthesis through the elevation of the mitochondrial superoxide levels [93,94]. Both compounds have shown allelopathic activity and suppressed the germination and growth of several plant species [122,123,128]. In addition, a mixture of volatiles, including precocenes I and II and three sesquiterpenes, namely β-caryophyllene, β-bisabolene, and β-farnesene, emitted from *Ageratum conyzoides*, may work as HIPVs involved in the indirect defense function against herbivore insects [67] (Figure 6).

Pyrrolizidine alkaloids, such as lycopsamine and echinatine, are highly toxic and possess insecticidal activity through the interruption of metabolism in insect cells, resulting in protection from herbivore insect attacks. These compounds may also work as defense agents and be involved in nematicidal activity. Flavonoids, such as nobiletin, 5′-methoxynobiletin, eupalestin, 5,6,7,3′,4′,5′-hexamethoxyflavone, and 5,6,8,3,4′,5′-hexamethoxyflavone, found in *Ageratum conyzoides* were reported to show inhibitory activity regarding the spore germination of pathogenic fungi [91,92]. Benzoic acid, protocatechuic acid, gallic acid, *p*-coumaric acid, *p*-hydroxybenzoic acid, and ferulic acid may act as allelopathic agents, causing the inhibition of the germination and growth of other plant species [124,125,126]. The sesquiterpene β-caryophyllene and the flavonoids found in *Ageratum conyzoides* may also act as allelopathic agents [128] (Figure 6).

Pharmacological and phytochemical investigations have shown that *Ageratum conyzoides* contains many other secondary metabolites in several chemical classes, such as monoterpenes, sesquiterpenes, flavonoids, and sterols. Some of these compounds are related to pharmacological activity, such as anti-pyretic, anti-inflammatory, cardiovascular, and analgesic activity, which is exploited in medicinal treatment, and anti-microbial activity, which benefits food security [94,148,149,150,151,152,153]. Although the identified compounds have not yet been related to the invasiveness of *Ageratum conyzoides*, some of them may act as defense molecules for unknown functions.

In conclusion, the defense responses of invasive plants to their natural enemies, such as herbivores and pathogens, is one of the essential functions for their naturalization and population expansion in their introduced ranges. The allelopathy of invasive plants against indigenous plant species is also one of these functions. As described above, *Ageratum conyzoides* produces several compounds that act as defense molecules against its natural enemies, such as herbivore insects, parasitic nematodes, and fungal pathogens, and act as allelochemicals against neighboring plant species. Therefore, these compounds may contribute to the naturalization and expanding population of *Ageratum conyzoides* in new habitats as an invasive plant species. These compounds may be used in the development of insecticides, fungicides, and/or herbicides.

## Data Availability

Not applicable.
